# Colorectal cancer-associated fibroblasts promote metastasis by up-regulating LRG1 through stromal IL-6/STAT3 signaling

**DOI:** 10.1038/s41419-021-04461-6

**Published:** 2021-12-20

**Authors:** Beiping Zhong, Bing Cheng, Xiaoming Huang, Qian Xiao, Zhitong Niu, Yu-feng Chen, Qiang Yu, Wenyu Wang, Xiao-Jian Wu

**Affiliations:** 1 Department of Colorectal Surgery, Guangdong Provincial Key laboratory of Colorectal and Pelvic Floor Disease, The Sixth Affiliated Hospital, Sun Yat-sen University, Guangzhou, 510655 China; 2 Guangdong Research Institute of Gastroenterology, The Sixth Affiliated Hospital, Sun Yat-sen University, Guangzhou, 510655 China; 3 Department of Hepatobiliary Surgery, the Sixth Affiliated Hospital, Sun Yat-Sen University, Guangzhou, 510655 China; 4 Cancer Precision Medicine, Genome Institute of Singapore, Agency for Science, Technology, and Research, Biopolis, Singapore, 138672 Singapore; 5 Department of Physiology, Yong Loo Lin School of Medicine, National University of Singapore, Singapore, 117597 Singapore; 6 Cancer and Stem Cell Biology, DUKE-NUS Graduate Medical School of Singapore, Singapore, 169857 Singapore

**Keywords:** Colon cancer, Cancer microenvironment

## Abstract

Cancer-associated fibroblasts (CAFs) have been shown to play a strong role in colorectal cancer metastasis, yet the underlying mechanism remains to be fully elucidated. Using CRC clinical samples together with ex vivo CAFs-CRC co-culture models, we found that CAFs induce expression of Leucine Rich Alpha-2-Glycoprotein 1(LRG1) in CRC, where it shows markedly higher expression in metastatic CRC tissues compared to primary tumors. We further show that CAFs-induced LRG1 promotes CRC migration and invasion that is concomitant with EMT (epithelial-mesenchymal transition) induction. In addition, this signaling axis has also been confirmed in the liver metastatic mouse model which displayed CAFs-induced LRG1 substantially accelerates metastasis. Mechanistically, we demonstrate that CAFs-secreted IL-6 (interleukin-6) is responsible for LRG1 up-regulation in CRC, which occurs through a direct transactivation by STAT3 following JAK2 activation. In clinical CRC tumor samples, LRG1 expression was positively correlated with CAFs-specific marker, α-SMA, and a higher LRG1 expression predicted poor clinical outcomes especially distant metastasis free survival, supporting the role of LRG1 in CRC progression. Collectively, this study provided a novel insight into CAFs-mediated metastasis in CRC and indicated that therapeutic targeting of CAFs-mediated IL-6-STAT3-LRG1 axis might be a potential strategy to mitigate metastasis in CRC.

## Introduction

Colorectal cancer (CRC) is the third most common malignancy and also the third leading cause of cancer-related death. Liver metastasis has been the most frequent and predominant reason accounting for patient mortality, with an overall 5-year survival rate less than 20% [1–3]. However, the underlying mechanisms of CRC metastasis have not been fully understood yet. Hence, a better understanding of molecular mechanism leading to metastasis is of urgent need and critical to improve metastatic patients’ survival.

According to a recently available classification system, CRC can be divided into four consensus molecular subtypes (CMSs), by which CMS4 is characterized by prominent transforming growth factor β activation and stromal invasion. Moreover, CMS4 is more aggressive and metastatic than other CMSs, indicating an important role of stromal cells in mediating metastasis in CRC [4–7].

Cancer-associated fibroblasts (CAFs), the most abundant component in the stroma, have been implicated in modulating tumor progression and therapeutic response [8]. A recent delicate study using single-cell technology combined with high-content digital imaging has demonstrated that the abundance of CAFs was linked with cancer heterogeneity and invasive potential in pancreatic cancer [9]. Consistently, many studies have elucidated that secreted factors derived from CAFs such as exosomes, non-coding RNA and cytokines act as the “intermediates” for CAFs to crosstalk with cancer cells and exert their functions [10–13]. With the unraveling relationship between cancer cells and CAFs, CAFs and/or its secretome are now being considered as potential targets for anti-tumor therapy [14].

Leucine Rich Alpha-2-Glycoprotein 1(LRG1) was firstly identified as an inflammatory protein in human serum by Haupt and Baudner in 1977 [15], but with little attention paid until 2013, when Wang et al reported that LRG1 could promote angiogenesis by modulating endothelial transforming growth factor β (TGFβ) signaling [16]. Since then, accumulating studies have focused on unveiling the potential roles of LRG1 in regulating tumor progression. Overexpression of LRG1 has been found in multiple cancers such as pancreatic ductal adenocarcinoma (PDAC) and ovarian cancer, where it promotes cell proliferation, migration and invasion. In accordance, serum LRG1 was significantly increased in PDAC and correlated with progressive clinical stage [17–19]. There are also contradictory findings showing that LRG1 suppresses migration and invasion of esophageal squamous cell carcinoma (ESCC) and hepatocellular carcinoma (HCC) [20, 21]. Thus, further studies are warranted to determine the exact role of LRG1 in tumor progression in a context-dependent manner. Moreover, whether CAFs could facilitate migration and invasion via regulating LRG1 remains unclear.

Using the in vitro CAFs-cancer cell co-culture model system, clinical samples from primary and liver metastatic patients, and high-content RNA-seq analysis, we identified that in the present work, LRG1 is not only highly expressed in metastatic lesions, but also steadily upregulated upon co-culture with CAFs. Further experiments demonstrated that CAFs induced EMT and promoted tumor migration and invasion by upregulation of LRG1 in cancer cells through the IL-6-STAT3 axis. Moreover, additional analysis indicated that LRG1 was a direct transcriptional target of STAT3. In accordance, blocking CAFs-LRG1 cascade was able to attenuate migrative and invasive capabilities of cancer cells, and liver metastasis in mouse model. More importantly, the expression level of α-SMA, a classical CAFs marker, was positively correlated with LRG1, and high expression of LRG1 predicted poor outcomes in the clinic. Overall, our study uncovered a novel mechanism by which CAFs mediated tumor metastasis and provides a potential strategy for tackling metastatic CRC patients.

## Results

### LRG1 is upregulated in metastatic colorectal cancer, which is associated with cancer-associated fibroblasts

To explore the critical mechanisms underlying CRC metastasis promoted by cancer-associated fibroblasts, we firstly isolated CAFs from primary tumors of CRC patients and normal fibroblasts (NFs) from the adjacent normal mucosa of CRC patients as previously reported [22]. Phase-contrast microscopy revealed the typical spindle-like morphology of both CAFs and NFs as expected (Supplementary Fig. 1a). Furthermore, CAFs (CAF1, CAF2) showed higher expression of specific markers including a-SMA, fibronectin, and P4HA1 [8], compared with NFs (NF1, NF2) and cancer cells (DLD-1, HCT-116), indicating the successful establishment of CAFs (Supplementary Fig. 1b).

**Fig. 1:**
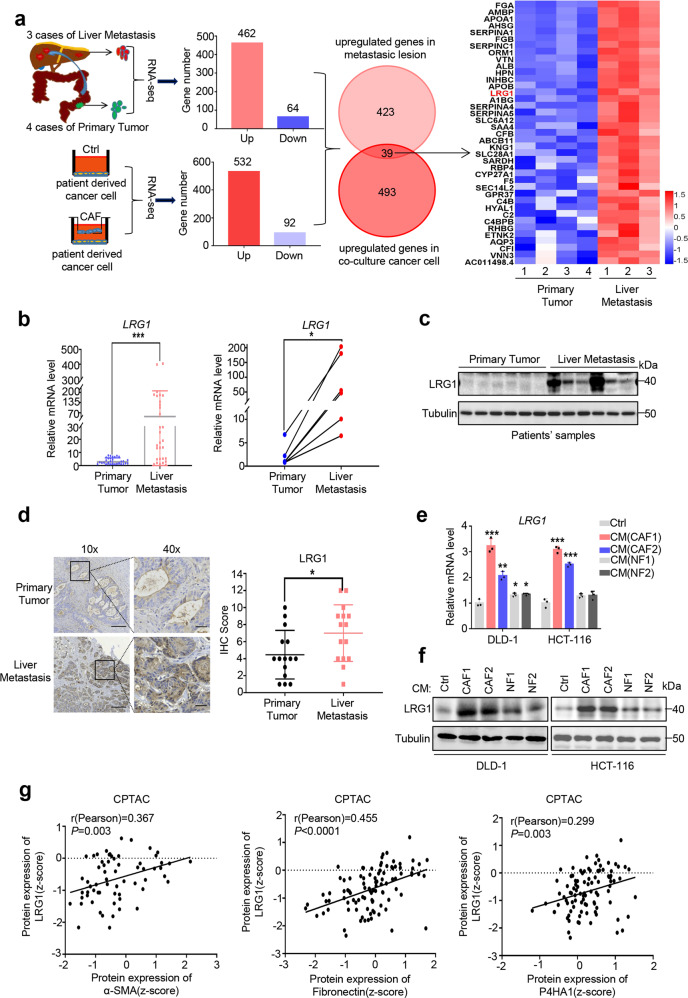
LRG1 is up-regulated in metastatic colorectal cancer which is associated with cancer-associated fibroblasts. **a** Scheme depicting the experimental design to search for genes involved in metastasis of CRC which can be induced by CAFs. Heatmap showing 39 hits up-regulated in liver metastatic lesions after overlapping. log2 fold change >2 and false discovery rate [FDR] < 0.01 were set as cut-off. **b** QRT-PCR analysis of LRG1 expression in tumor sections from primary and liver metastatic CRC patients. Left: unpaired tumor samples, *n* = 30 primary tumors and *n* = 30 liver metastases; Right: *n* = 6 paired tumor samples. **c** Western blot analysis of LRG1 expression in tumor sections from primary and liver metastatic CRC patients (*n* = 6 unpaired tumor samples). Blots were probed against LRG1 and β-tubulin. **d** Representative IHC staining of LRG1 in tumor sections from primary and liver metastatic CRC patients (Scale bar, 50 μm). Plot showing IHC score of LRG1 staining intensity in tumor species from primary and liver metastatic CRC patients (*n* = 15). **e**, **f**. QRT-PCR and western blot analysis of LRG1 expression in DLD-1 and HCT-116 in presence or absence of conditioned medium (CM) from two individual CAF and NF. **g** Correlation analysis of α-SMA/Fibronectin/P4HA1 protein level with that of LRG1 in CRC primary tumors using CPTAC database. Error bars represent SD; *n* = 3. *P < 0.05, **P < 0.01, ***P < 0.001, ****P < 0.0001.

Next, we sought to figure out the critical genes in tumor cells associated with CAF-driven metastasis. To this end, we performed RNA sequencing (RNA-seq) analysis of patient-derived cancer cells with or without co-culture with CAFs together with 4 primary tumors and 3 liver metastases from CRC patients. Overlapping transcriptomic analysis revealed that 39 genes were up-regulated in both cancer cells co-cultured with CAFs and metastatic tumors compared to primary tumors (cut-off of FDR < 0.01 and log_2_ fold change>2) (Fig. 1a). Among these genes, LRG1 has attracted our attention since it ranked as the top hits and has recently been reported to be associated with tumor recurrence in CRC [23], though the underlying mechanism was unknown (Fig. 1a). Previous work has reported that hepatocytes are one of the sources of LRG1 [24]. To exclude the possible inaccurate conclusion due to hepatocytes contamination in the metastatic tumor specimens, we performed IHC staining of LRG1 and confirmed that tumor cells are the major component which expressed a comparable level of LRG1 to hepatocytes (Supplementary Fig. 2a). To validate RNA sequencing data, 60 unpaired primary (*n* = 30) and liver metastatic CRC (*n* = 30) in addition to 6 paired primary and liver metastatic CRC samples were subjected to quantitative reverse transcription PCR (qRT-PCR) analysis, and the data confirmed a significant increase of LRG1 in the LM lesions (Fig. 1b). Furthermore, western blot (WB) and immunohistochemistry (IHC) analysis of tissues from primary and metastatic tumors also verified the upregulation of LRG1 in the liver metastatic lesions (Fig. 1c and d). LRG1 overexpression in metastasis compared to primary tumors was also observed in two publicly available databases (GSE14297, GSE50760) (Supplementary Fig. 2b, c). Of note, LRG1 was also found to be highly expressed in primary tumors compared to normal tissue, indicating a potential pro-tumorigenic role of LRG1 in CRC (Supplementary Fig. 2d, GSE20842).

To further verify the role of CAFs in regulating LRG1 expression, CRC cancer cell lines, DLD-1 and HCT-116, were co-cultured with conditioned medium (CM) from either CAFs or NFs and LRG1 expression was subsequently tested by qRT-PCR and WB. Not surprisingly, consistent with our aforementioned RNA-seq analysis, CM-derived from CAFs was able to robustly induce LRG1 expression, whilst CM-derived from NFs only showed mild induction of LRG1(Fig. 1e, f). Moreover, we interrogated data from CPTAC (clinical proteomic tumor analysis consortium) and performed correlation analysis. Intriguingly, as speculated, LRG1 expression has shown a significantly positive correlation with CAFs markers including α-SMA (Pearson’s r = 0.367; *p* = 0.003), fibronectin (Pearson’s r = 0.455; *p* < 0.0001), P4HA1 (Pearson’s r = 0.299; *p* = 0.003). This analysis strongly suggested the association of CAFs with LRG1 in clinical CRC samples (Fig. 1g). Collectively, these results support LRG1 as an oncogene involved in metastasis of CRC and CAFs contribute to its up-regulation in CRC.

### CAFs-induced LRG1 promotes CRC migration and invasion

**Fig. 2:**
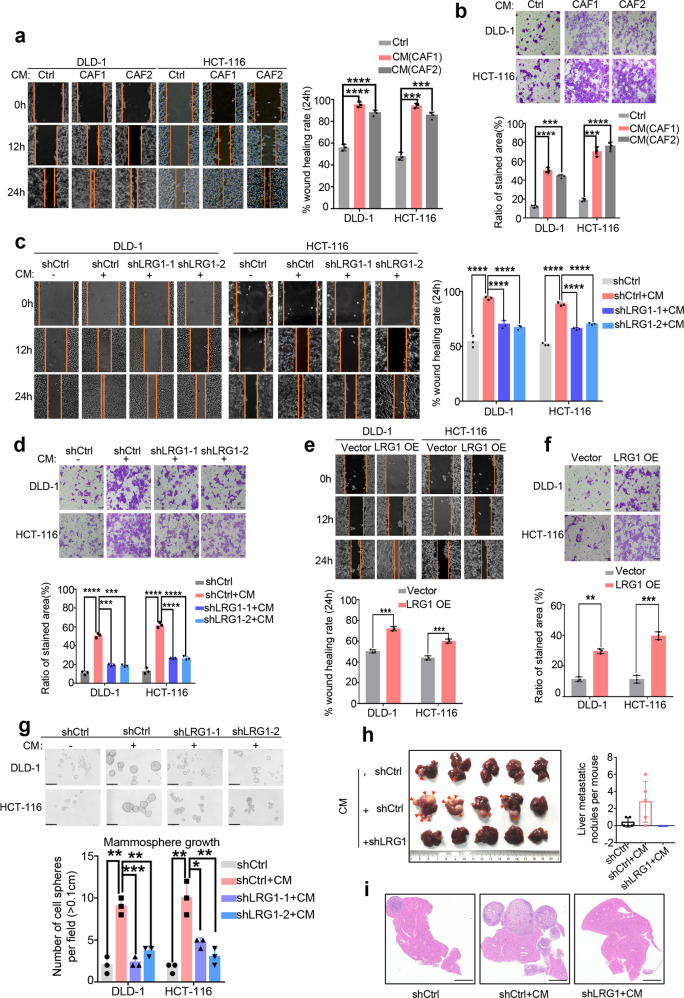
Cancer-associated fibroblasts promote cell migration and invasion in a LRG1-dependent way. **a** Migratory ability of DLD-1 and HCT-116 upon co-culture with CM from CAFs or not was assessed by scratch wound healing assay. Left: Representative images of CM-treated DLD-1 and HCT-116 taken at the indicated time intervals. Right: Plot showing wound healing rate of DLD-1 and HCT-116 upon co-culture with CM from CAFs or not at 24 h. **b** Invasive capability of DLD-1 and HCT-116 with or without CM treatment was assessed by Boyden chamber trans-well assay. Up: Representative images of CM-treated DLD-1 and HCT-116 taken at the indicated time intervals (Scale bar, 50 μm). Down: Plot showing the percentage of invasive cells area upon co-culture with CM or not at 24 h. **c** Migratory capability of DLD1 and HCT-116 induced by CM in presence or absence of LRG1 was assessed by scratch wound healing assay. Hereafter, CM represents conditioned medium from CAF1 unless stated otherwise. Left: Representative images of CM-treated DLD-1 and HCT-116 with or without silencing of LRG1. Right: Plot showing statistical analysis of wound healing rate at 24 h. **d** Invasive capability of DLD1 and HCT-116 induced by CM in the presence or absence of LRG1 was assessed by Boyden chamber assay. Up: Representative images of CM-treated DLD-1 and HCT-116 with or without silencing of LRG1 (Scale bar, 50 μm). Down: Plot showing statistical analysis of the percentage of invasive cells area at 24 h. **e** Migrative ability of DLD-1 and HCT-116 with ectopic expression of LRG1 or not was analyzed by scratch wound healing assay. Up: Representative images of DLD-1 and HCT-116 with or without ectopic expression of LRG1. Down: Plot showing rate of wound healing in DLD1 and HCT-116 with or without LRG1 overexpression at 24 h. **f** Invasive capability of DLD-1 and HCT-116 with ectopic expression of LRG1 was assessed by Boyden chamber assay. Up: Representative images showing invaded DLD-1 and HCT-116 cells (Scale bar, 50 μm). Down: Graphs showing statistical analysis of the percentage of invasive cells area at 24 h. **g** Tumorsphere growth of DLD1 and HCT-116 induced by CM in the presence or absence of LRG1 was assessed by tumorsphere formation assay after 7 days culture. Up: Representative images showing spheroids of DLD-1 and HCT-116 cells (Scale bar, 50 μm). Down: Graphs showing statistical analysis of the number of spheroids with diameter >0.1 cm. **h** Left: Images showing the liver metastasis of DLD-1 cells which expressed indicated shRNAs and were cultured with control medium or conditioned medium from CAFs before transsplenic injection into nude mice. (The red arrows indicate metastatic nodules). Right: Plot showing the liver metastatic nodules per mouse in three groups. **i** Representative images showing H&E staining of liver tissue samples from the three groups as indicated in h (Scale bar, 50μm). Error bars represent SD; *n* = 3. **p < 0.01, ***p < 0.001, ****p < 0.0001.

Having shown that CAFs induce expression of LRG1 in metastatic CRC and display a positive correlation with LRG1 expression in clinic, we next sought to determine if this activity is required for migration and invasion of CRC. To achieve this, we first performed scratch wound healing, and Boyden chamber trans-well assays in CRC treated with or without CAFs-derived conditioned medium (CM). Although conditioned medium from CAF did not promote cell proliferation within 48 h (Supplementary Fig. 3), we observed a notable increase in migration and invasion induced by CAFs-CM at 24 h, supporting the intact pro-metastatic property of CAFs established in our lab (Fig. 2a, b). Moreover, silencing of LRG1 with two independent shRNA significantly impeded CAF-CM-induced migration of DLD-1 and HCT-116 (Fig. 2c and Supplementary Fig. 4a). Similarly, knockdown of LRG1 was able to attenuate CAFs-induced invasion (Fig. 2d). Conversely, ectopic expression of LRG1 in DLD-1 and HCT-116 in the absence of CAFs-CM was sufficient to induce migration and invasion (Fig. 2e, f and Supplementary Fig. 4b), but not cell proliferation (Supplementary Fig. 5). In addition, we assessed the effect of CM-induced LRG1 on tumorsphere formation using DLD1 and HCT-116 cells which expressed control or *LRG1* shRNA respectively. As expected, conditioned medium from CAF resulted in robust sphere formation while silencing of LRG-1 attenuated this effect remarkably (Fig. 2g). In line with in vitro analysis, the splenic liver metastatic mouse model showed conditioned medium pretreated DLD-1 cells were not only able to form more but also bigger metastatic nodules in liver compared to cells treated with control medium. Intriguingly, inhibition of LRG-1 exhibited almost absolute abolishment of the metastatic nodules formation. (Fig. 2h, i; Supplementary Fig. 6a, b). Together, these consistent findings strongly indicated that CAF-mediated induction of LRG1 played a crucial role in driving CRC migration, invasion and liver metastasis.

### CAFs promotes EMT in a LRG1-dependent manner

**Fig. 3:**
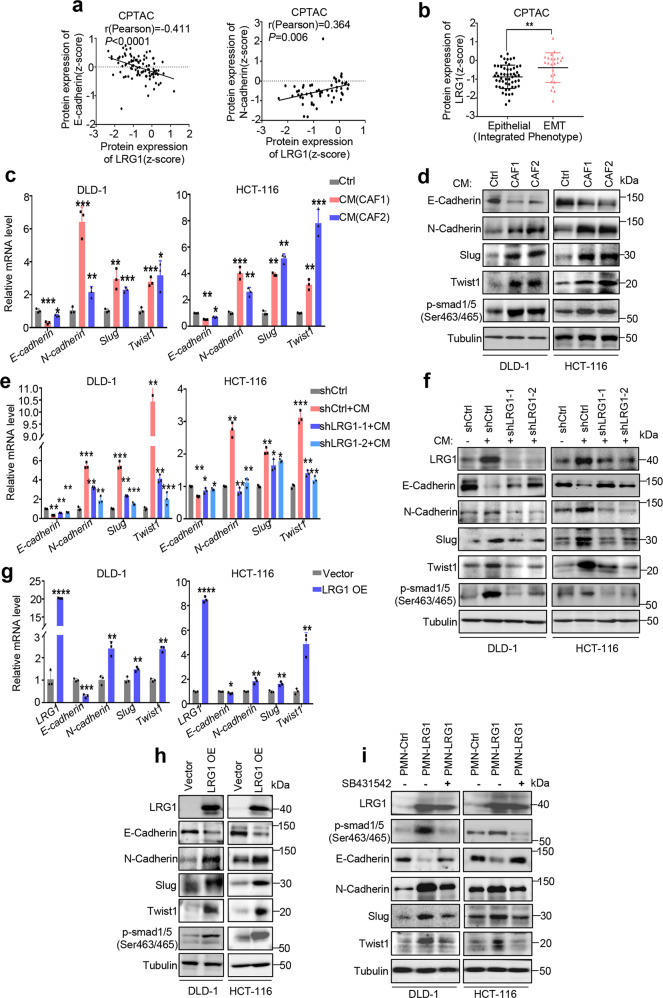
CAFs-induced LRG1 promotes EMT. **a** Correlation analysis between E-cadherin/N-cadherin protein expression and LRG1 expression in CRC primary tumors in CPTAC database. **b** Graph showing LRG1 protein expression in CRC primary tumor with two different integrated phenotypes (epithelial versus EMT) based on data from CPTAC. **c** EMT associated markers were analyzed by qRT-PCR in DLD-1 and HCT-116 upon treatment with control medium or CM from two individual CAF. **d** EMT associated markers and phosphorylated Smad1/5(ser463/465) were analyzed by western blot in DLD-1 and HCT-116 upon treatment with control medium or CM from two individual CAF. **e** DLD-1 and HCT-116 with or without silencing of LRG1 were treated with control medium or CM from CAF. Expression of EMT-associated markers was measured by qRT-PCR. **f** DLD-1 and HCT-116 with or without silencing of LRG1 were treated with control medium or CM from CAF. Expression of EMT-associated markers and phosphorylated Smad1/5(ser463/465) was measured by western blot. **g** Expression of EMT-associated markers was measured by qRT-PCR in DLD-1 and HCT-116 with or without ectopic expression of LRG1. **h** Expression of EMT-associated markers and phosphorylated Smad1/5(ser463/465) was measured by western blot in DLD-1 and HCT-116 with or without ectopic expression of LRG1. **i** DLD1 and HCT-116 in the presence or absence of LRG-1 overexpression were pre-treated with SB431542, a TGFβ-Smad pathway inhibitor, or not. Expression of LRG1, phosphorylated Smad1/5 (ser463/465) and EMT-associated markers were measured by western blot. Error bars represent SD; *n* = 3. *P < 0.05, **P < 0.01, ***P < 0.001, ****P < 0.0001.

As EMT (epithelial-mesenchymal transition) was considered as a cellular program which has been implicated in carcinogenesis and enhanced metastatic properties of cancer cells [25, 26], correlation analysis of LRG1 and two typical EMT markers including epithelial marker E-cadherin and mesenchymal marker N-cadherin was firstly performed using CPTAC database. As expected, the data showed that LRG1 was negatively correlated with epithelial marker E-cadherin (Pearson’s r = 0.411; *p* < 0.0001) and positively correlated with mesenchymal marker N-cadherin (Pearson’s r = 0.364; *p* = 0.006) (Fig. 3a). In addition, analysis of LRG1 expression levels in two integrated phenotypes of colorectal cancer patients which were defined by epithelial or mesenchymal phenotype associated gene signatures indicated that LRG1 was significantly enriched in tumor tissues with EMT status [27, 28] (Fig. 3b). To confirm the role of LRG1 in modulating EMT, we further examined several markers associated with EMT, including E-cadherin, N-cadherin, Slug, Twist1 in CRC cells with knockdown of LRG1 or overexpression of LRG1. Consistent with that CAFs-derived CM can induce migration and invasion, we showed that CRC cells treated with CAFs-derived CM also exhibited an evident increase of mesenchymal markers and decreased the epithelial markers, indicating EMT promotion by CAFs (Fig. 3c, d). Interestingly, phosphorylation of Smad1/5 at Ser463/465, which has been implicated in epithelial-mesenchymal transition [29, 30], was obviously induced in cancer cells by CAFs-derived CM, suggesting CAFs might be able to exert pro-metastatic role via LRG1-mediated activation of Smad1/5 signaling (Fig. 3d). To test this, we performed LRG1 knockdown in cancer cells when cultured with CAF conditioned medium. As anticipated, silencing of LRG1 was able to dramatically impair CAFs-induced EMT accompanied by decreased phosphorylation of Smad1/5 in cancer cells (Fig. 3e, f). Conversely, overexpression of LRG1 was sufficient to induce EMT along with activation of Smad1/5 signaling in the absence of CAF-CM (Fig. 3g, h). To further dissect the role of Smad1/5 phosphorylation in LRG1-induced EMT, analysis of EMT associated markers as well as invasive ability was performed in the presence or absence of SB431542, a selective TGFBR1/ACVR1B/ACVR1C inhibitor which could sufficiently inhibit Smad1/5 phosphorylation. Not very surprisingly, suppression of Smad1/5 was indeed able to largely reverse LRG1-induced EMT and invasion accordingly (Fig. 3i and Supplementary Fig. 7a). Recently, STAT3 signaling was also reported to be involved in LRG1-promoted metastatic dissemination of melanoma [31]. Whereas, p-STAT3 blockade by pacritinib showed little effect on LRG1-induced EMT in CRCs (Supplementary Fig. 7b) indicating diverse downstream signaling pathways involved in LRG-1 mediated metastasis in a context dependent manner. Taken together, these analyses supported that LRG1 plays an important role in CAFs-induced EMT in CRC, which might depend on LRG1-related Smad1/5 activation.

### CAFs-secreted IL-6 up-regulates LRG1 through activating JAK2/STAT3 signaling

**Fig. 4:**
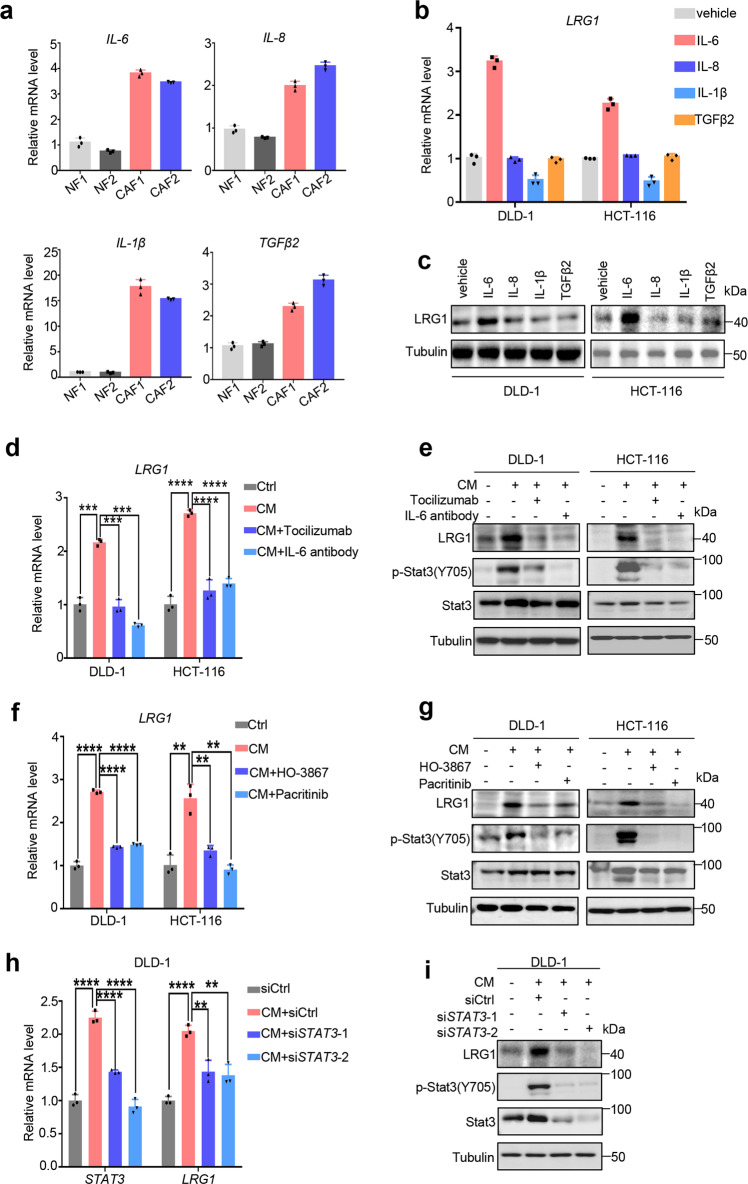
CAFs-secreted IL6 up-regulates LRG1 through activating JAK2/STAT3 signaling. **a** QRT-PCR analysis of IL-6/IL-8/IL-1β/TGFβ2 mRNA level in CAFs compared to NFs. **b** & **c**. QRT-PCR and western blot analysis of LRG1 expression in DLD-1 and HCT-116 treated with vehicle or recombinant IL-6/IL-8/IL-1β/TGFβ2. **d**, **e**. DLD1 and HCT-116 were cultured with control medium, CM from CAF1 or treated with neutralizing antibody either targeting IL-6R or IL-6 when cultured with CM from CAFs as indicated. Expression of LRG1 was measured by qRT-PCR and western blot. Blots were probed against LRG1, phosphorylated STAT3(Y705), total STAT3 and β-tubulin. **f**, **g** DLD1 and HCT-116 were cultured with control medium, CM from CAFs or treated with HO-3867 (STAT3 inhibitor) or pacritinib (JAK2 inhibitor), when cultured with CM from CAFs as indicated. Expression of LRG1 was subsequently analyzed by qRT-PCR and western blot. Blots were probed against LRG1, phosphorylated STAT3(Y705), total STAT3 and β-tubulin. **h**, **i** DLD1 was transfected with control siRNA or two independent siRNA targeting STAT3, followed by culturing with control medium or CM from CAFs. Expression of LRG1 and STAT3 was measured by qRT-PCR and western blot. Blots were probed against LRG1, phosphorylated STAT3(Y705), total STAT3 and β-tubulin. Error bars represent SD; *n* = 3. **P < 0.01, ***P < 0.001, ****P < 0.0001.

As shown above, CM from CAFs was sufficient to promote LRG1 expression and EMT in CRC, which indicated CAFs-secreted factors might play a significant role in regulating LRG1 expression. Several studies including ours reported that CAFs-secreted cytokines including IL-6, IL-8, TGF-β, and IL-1β participated in tumor progression through holding communication between CAFs and cancer cells [32–35]. First, we examined the expression of these cytokines in our established CAFs. In line with previous studies, CAFs show higher expression of all four cytokines compared to NFs (Fig. 4a). To further dissect which cytokine plays a dominant role in mediating LRG1 upregulation, we treated two CRC cancer cell lines, DLD-1 and HCT-116 with four recombinant cytokines separately, and measured LRG1 expression by qRT-PCR and WB. Among these cytokines, only recombinant IL-6 was able to significantly induce LRG1 expression in cancer cells (Fig. 4b, c). Conversely, adding neutralizing antibody either targeting IL-6 or IL-6R could efficiently block the induction of LRG1 mediated by CAFs, supporting that IL-6 was the essential effector derived from CAFs to promote LRG1 expression in cancer cells (Fig. 4d, e). Since IL-6 has been reported to stimulate the activation of JAK2/STAT3 signaling and such aberrant activation was generally associated with poor clinical prognosis, we assumed that upregulation of LRG1 in cancer cells might be attributed to activation of JAK2/STAT3 axis induced by CAFs-secreted IL-6. To this end, two CRC cancer cell lines, DLD-1 and HCT-116, were pre-treated with pacritinib or HO-3867 which are extensively investigated clinical or pre-clinical inhibitors targeting JAK2 and STAT3 respectively and subsequently co-cultured with conditioned medium from CAFs. The results demonstrated that the two inhibitors could successfully inhibit activation of JAK2/STAT3 signaling indicated by reduction in phosphorylation of STAT3 at Tyr 705. And concomitantly, blockade of JAK2/STAT3 signaling markedly decreased expression of LRG1 induced by CAFs both at mRNA and protein levels (Fig. 4f, g). Consistent with this, direct interference of STAT3 by siRNA also profoundly inhibited LRG1 expression induced by CAFs (Fig. 4h, i), strongly indicating JAK2/STAT3 cascade was indeed involved in IL-6 mediated LRG1 up-regulation. Taken together, these data unequivocally confirmed that CAFs-secreted IL-6 induced LRG1 expression through downstream activation of JAK2/STAT3 signaling in cancer cells.

### Stat3 transactivates LRG1 expression through directly binding to its promoter

**Fig. 5:**
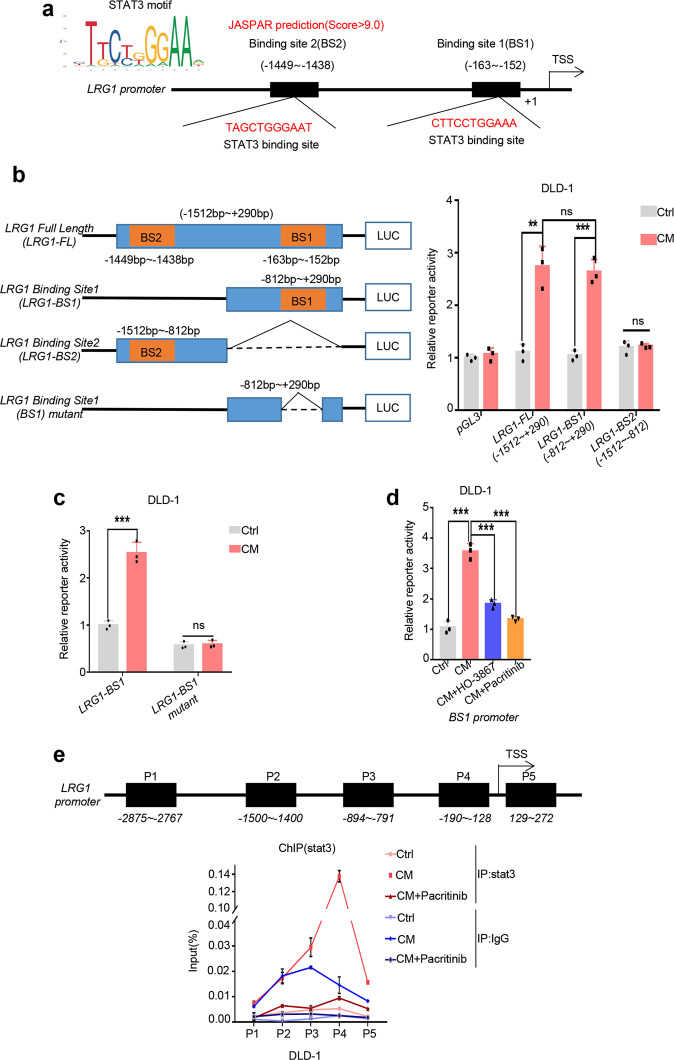
STAT3 regulates LRG1 expression through direct binding to its promoter. **a** Schematic illustration of potential STAT3 binding sites located at LRG1 promoter region predicted by JASPAR. **b** Left: Scheme diagram depicting the design of LRG1 promoter regions in the luciferase reporter plasmids. Right: Activity of different fragments of LRG1 promoter in response to CM from CAF1 was determined by dual-luciferase assay. **c** Activity of LRG1 promoter with deletion of BS1 or not (named LRG1-BS1 and LRG1-BS1 mutant respectively) was tested by dual-luciferase assay. Graph showing relative activity of either promoter upon co-culture with CM from CAF1 or not. **d** DLD1 with LRG1 promoter containing BS1 was pre-treated with HO-3867 or pacritinib, followed by incubation with CM from CAF1 or not. Activity of LRG1 promoter was analyzed by dual-luciferase assay. **e** Top: Scheme depicting the regions where primers were designed for amplifying across LRG1 promoter. Bottom: DLD1 was pre-treated with or without pacritinib, followed by co-culture with CM from CAF1 or not. Quantitative ChIP-PCR assay was performed to detect the occupancy of stat3 protein at the different regions of the LRG1 promoter under indicated conditions. Error bars represent SD; *n* = 3. **P < 0.01, ***P < 0.001, ****P < 0.0001; ns for no significant.

STAT3 has been described as a master transcriptional factor that drives tumor progression of multiple cancers via regulating the expression of key target genes such as VEGF, MMP and IL-6 [36–38]. As mentioned above, upregulation of LRG1 in cancer cells relied on JAK2/STAT3 signaling, we hypothesized that LRG1 could be a potential novel target regulated by STAT3. To address this question, in silico analysis of LRG1 promoter region was firstly performed using JASPAR database and we found that there were two consensus STAT3 binding sites located at −163~−152 and −1449~−1438 with score >9.0 respectively, raising the possibility that LRG1 might be a direct transcriptional target of STAT3 (Fig. 5a). To verify this, we constructed plasmids containing full-length LRG1 promoter region or two truncated forms with depletion of either STAT3 binding sites (hereinafter referred to as LRG1-FL, LRG1-BS1 and LRG1-BS2) and dual-luciferase based reporter assay was performed. Both LRG1-FL and LRG1-BS1 were responsive to CAF-derived conditioned medium treatment, while LRG1-BS2 failed to show any response, suggesting a pivotal role of BS1 in regulation of LRG1 stimulated by CAFs (Fig. 5b). In addition, to exclude possible influence of other transcriptional factors regulated by CAFs, we further precisely deleted the binding site of STAT3 located on LRG1-BS1 (LRG1-BS1mutant) and performed dual-luciferase based reporter assay. As expected, deletion of STAT3 binding site could completely abrogate the response of LRG1-BS1 to CAF-derived conditioned medium treatment (Fig. 5c). Moreover, pre-treatment with HO-3867 and pacritinib, inhibitors targeting JAK2/STAT3 cascade, could successfully repress LRG1-BS1 reporter activity induced by CAFs, supporting that CAFs-mediated regulation of LRG1 was JAK2/STAT3 dependent (Fig. 5d). In accordance, chromatin immunoprecipitation (ChIP) assay was carried out to determine the direct recruitment abundance of STAT3 at LRG1 promoter. Consistent with reporter assay, activated STAT3 was predominantly bound to BS1 upon treatment with conditioned medium from CAFs and this binding could be dampened after inhibition of JAK2/STAT3 activity by pacritinib (Fig. 5e). Collectively, these findings suggested that LRG1 is a direct transcriptional target of STAT3.

### CAFs-associated high expression of LRG1 predicts poor clinical outcome in CRC

**Fig. 6:**
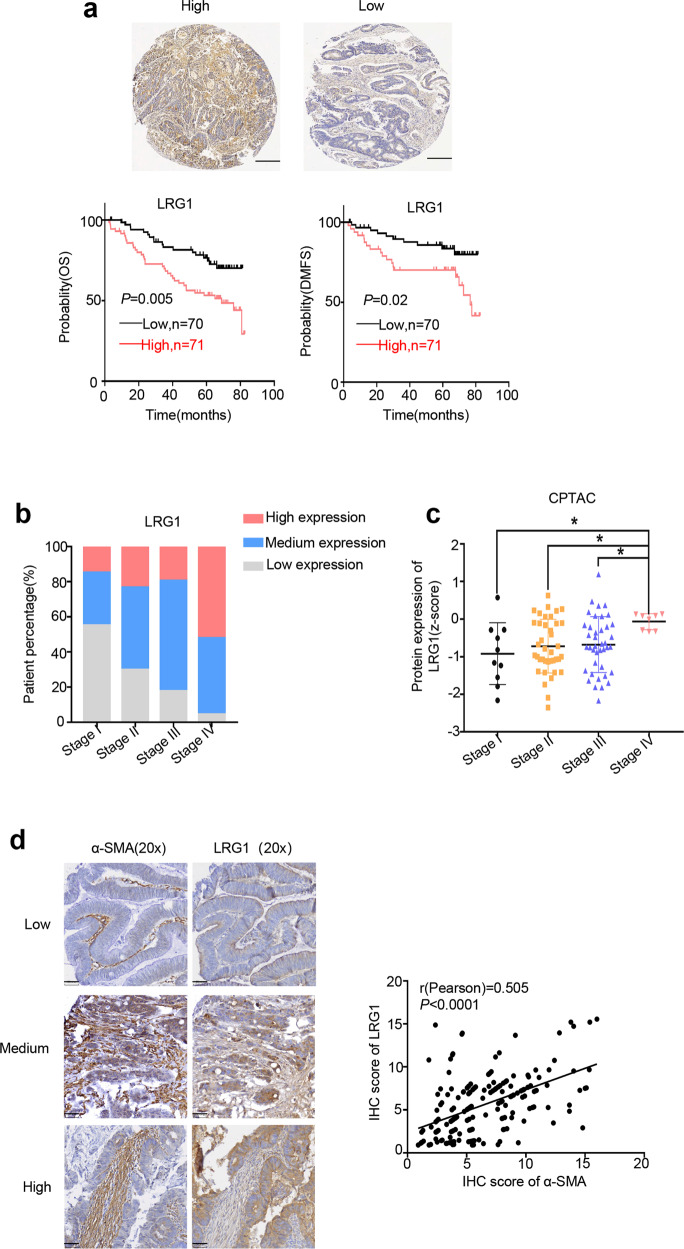
CAFs-associated high expression of LRG1 predicts poor clinical outcome in CRC. **a** Up: Representative IHC staining of LRG1 in TMA (Scale bar, 100 μm). Down: Kaplan–Meier analysis of the association of overall survival (OS) or distant metastasis free survival (DMFS) with LRG1 protein level in 141 CRC patients from TMA. **b** Graph showing the percentage of patients with different level of LRG1 in four different stages in 141 CRC patients from TMA. I-IV represent stages of patients according to AJCC TNM staging system. (*n* = 20, stage I; *n* = 47, stage II; *n* = 51, stage III; *n* = 23, stage IV). **c** Graph showing LRG1 protein level in different TNM stages using data from CPTAC. **d** Left: Representative IHC staining of LRG1 and α-SMA in TMA (Scale bar, 50 μm). Right: Plot showing correlation between α-SMA and LRG1 protein level in patients from TMA (*n* = 190). Error bars represent SD; *n* = 3. *P < 0.01.

To evaluate the relevance between LRG1 and clinical outcome, we examined LRG1 expression by immunohistochemistry (IHC) in the tissue microarray (TMA) which consists of tumor specimens of 141 stage I-stage IV CRC patients with up to 5 years of follow-up information. Kaplan-Meier analysis revealed that patients with high LRG1 expression had poor prognosis compared to those with low LRG1 expression defined by significantly reduced overall survival (OS), and distant metastasis-free survival (DMFS) (Fig. 6a). In addition, as tumor progressed, percentage of patients with high LRG1 expression obviously increased, especially in patients with stage IV CRC (Fig. 6b) and this was further confirmed by bioinformatic analysis of data from CPTAC showing the protein level of LRG1 was highest in the tumors with the most advanced stage (Fig. 6c). Moreover, we also analyzed the correlation between LRG1 expression and several clinical pathologic factors. As shown in Table 1, LRG1 expression was positively correlated with tumor size, metastasis, invasion, and higher expression of the essential prognostic factor-CEA which also predicted worse clinical outcomes. These data collectively reinforce the clinical significance of LRG1. More importantly, to determine whether the CAFs-LRG1 axis does exist clinically, we further performed IHC analysis of a classic CAFs marker, α-SMA, and LRG1. As expected, α-SMA displayed positive correlation with LRG1 in clinical CRC samples (Fig. 6d). Together, these analyses highlight the clinical relevance of the newly identified CAFs-LRG1 regulatory axis.

**Table 1:** Correlation between LRG1 expression and clinicopathological parameters of CRC.

Clinicopathologic features	Number	LRG1 expression(IHC)		
		High	Low	*p* value
Gender				
Male	89	44	45	0.776
Female	52	27	25	
Age(years)				
<65	77	40	37	0.678
≥65	64	31	33	
Tumor size(cm)				
<5	19	5	14	0.024
≥5	122	66	56	
Degree of differentiation				
Highly	23	9	14	0.239
Moderately and poorly	118	62	56	
Depth of invasion				
T1 + T2	27	9	18	0.049
T3 + T4	114	62	52	
Lymphonodus metastasis				
N0	74	31	43	0.035
N1, N2	67	40	27	
Distance metastasis				
Negative	118	55	63	0.044
Positive	23	16	7	
Plasma CEA level (preoperative)				
≤5ng/ml	84	33	51	0.013
>5ng/ml	42	27	15	
Plasma CA19-9 level (preoperative)				
≤37u/ml	98	47	51	0.811
>37u/ml	21	9	12	

## Discussion

**Fig. 7:**
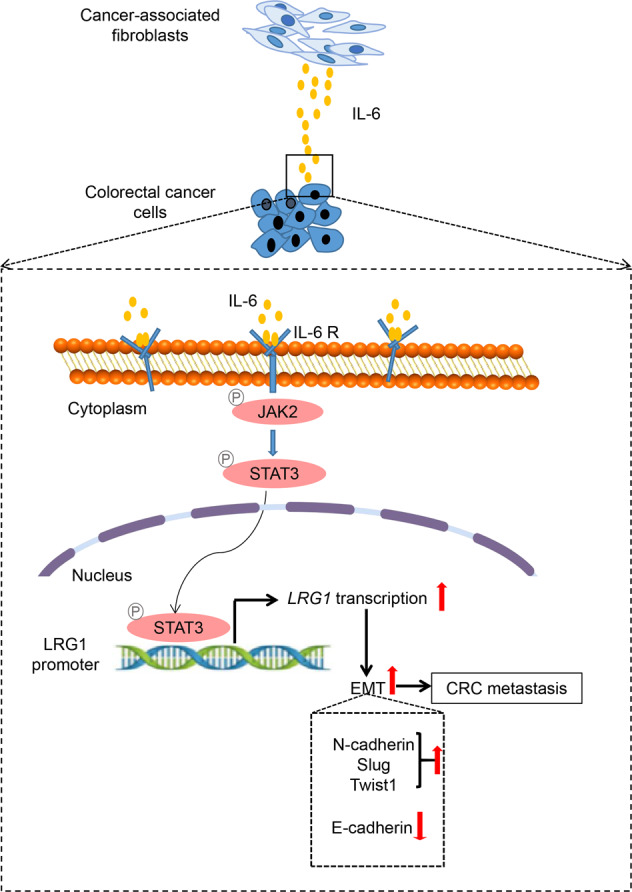
The proposed model for CAFs-derived IL-6/STAT3/LRG1 axis in promoting metastasis of CRC. Scheme diagram depicting the underlying mechanism involved in metastasis of CRC mediated by CAFs. CAFs-derived IL-6 is able to activate JAK2-STAT3 in cancer cells. As a master transcriptional factor, STAT3 directly binds to LRG1 promoter, thus leading to upregulation of LRG1. LRG1 further promotes migration, invasion as well as EMT which is crucial for CAFs-mediated metastasis of CRC.

In the current study, by taking advantage of high-content RNA-seq using clinical samples and in vitro co-culture system model, we have described a novel mechanism of metastasis by which CAFs promote migration and invasion via upregulation of LRG1 through the IL-6-STAT3 axis (Fig. 7). CAFs are the key component in the TME with diverse biological functions, including extracellular matrix remodeling and reciprocal interaction with other types of cells. The secretome of CAFs such as growth factors, cytokines and exosomes function as “messengers” to mediate crosstalk between cells. The interplay between CAFs and other cells in TME has been implicated in tumor progression regarding to modulating cell proliferation, invasion and therapeutic responses [39–41]. Our previous work has reported that CAFs-derived TGF-β2 could coordinate with HIF-1α under hypoxia to confer chemo-resistance via activation of GLI2 [22]. Here, intriguingly, our finding identified that IL-6 secreted by CAFs was able to up-regulate LRG1 expression to promote migration and invasion in CRC. Albeit exogenous supplement of TGF-βalone didn’t induce LRG1 expression, we still can’t preclude the potential role of TGF-βin regulating LRG1-mediated metastasis. It would be of interest to determine whether TGF-βwould be a prerequisite for LRG1 to exert its pro-metastatic function since TGF-βwas usually constitutively produced in the tumor milieu [42]. Furthermore, experiments revealed that the increase of LRG1 mediated by IL-6 depended on the activation of STAT3. Blockade of CAFs-LRG1 signaling cascade either by inhibitor or siRNA targeting JAK2/STAT3 axis can effectively attenuate migration and invasion of cancer cells induced by CAFs. This opens a new avenue to interfere with metastasis of CRC by targeting CAFs-derived IL-6-STAT3-LRG1 axis. More importantly, many of agents that target individual nodes, including IL-6, IL-6R, and JAKs/STAT3 are currently under active investigations as treatments of hematopoietic malignancies and solid tumors [43]. Undoubtedly, a deeper understanding of IL-6-STAT3 axis would benefit patients with therapies targeting this pathway.

Furthermore, in addition to direct effects on cancer cells, CAFs also regulate biological functions of other stromal cells such as immune cells. Albeit it is already well-established that CAFs can exert immunomodulating function via cytokine secretion, exclusion of anti-tumor immune cells from the tumor and/or recruitment of immunosuppressive immune cells to the tumors, the detailed molecular mechanisms underlying the regulation of immune response by CAFs are still unclear [44–46]. LRG1 is a secretory glycoprotein which is overexpressed in multiple cancers such as pancreatic, bladder and ovarian cancers. It is a highly conserved member of the leucine-rich repeat (LRR) family proteins, many of which have been commonly involved in cell signal transduction, cell adhesion, DNA repair and immune responses etc. [47]. In line with this, increased LRG1 has been observed in various inflammatory and autoimmune diseases [48–50]. Our work firstly identified a direct link of CAFs with LRG1 expression to promote CRC metastasis. Thus, it is of interest to explore whether the pro-metastatic role of LRG1 secreted by CAFs depends (at least partially) on its immunoregulatory function.

Although several studies implicated that LRG1 might be one of the chief culprits mediating tumor progression, the role of LRG1 in regulating metastasis remains largely unknown and seems to be controversial. Several groups reported an inhibitory role of LRG1 on the migrative and invasive potential of different tumors including hepatocellular carcinoma (HCC) and esophageal squamous cell carcinoma (ESCC) [20, 21], whereas the underlying mechanisms have not been fully explored yet Our work demonstrated that LRG1 was highly expressed in metastatic CRC tissues. This phenomenon was confirmed by testing clinical samples from our lab and can be extrapolated to more cohorts using public databases. In addition, silencing of LRG1 could significantly attenuate migratory and invasive property of CRC cells induced by CAFs and ectopic expression of LRG1 promotes migration and invasion respectively. More importantly, patients with higher expression of LRG1 indeed have lower overall survival and distant metastasis free survival rate, suggesting the important clinical relevance of LRG1 to metastasis of CRC. This evidence strongly supports the pro-metastatic role of LRG1 in CRC. However, how does one reconcile the fact that LRG1 can have multifaceted roles in regulating metastasis? We assume that this discrepancy might be attributed to complicated downstream signaling cascades activated by LRG1. Of note, our data showed that both CAFs and overexpression of LRG1 were capable of promoting phosphorylation of Smad1/5, and blockade of Smad1/5 signaling by inhibitor can effectively inhibit LRG1-induced EMT and invasion of cancer cells, indicating that activation of Smad pathway may be essential for metastasis induced by CAFs-LRG1 axis and further studies are warranted to address this question in depth.

In conclusion, our studies identified a novel signaling pathway by which CAFs mediate metastasis of CRC via up-regulation of LRG1 in an IL-6/STAT3 dependent manner. This work points to potential regimens targeting IL-6/STAT3/LRG1 axis in advanced CRC and LRG1 might be used as a powerful prognostic biomarker of metastatic CRC which is worthy of being further explored in the future.

## Materials and methods

### Culture of patient-derived fibroblasts

Human biological samples used to isolate fibroblasts were obtained from individuals treated at the Sixth Affiliated Hospital of Sun Yat-Sen University (Guangzhou, Guangdong, China), under informed consent and approval by the Ethics Committee of the Sixth Affiliated Hospital, Sun Yat-Sen University. All the studies with these samples were approved by IRB for research purposes. All CAFs (CAF1, CAF2) and NFs (NF1, NF2) are isolated from CRC patients. Among them, CAF1 or CAF2 was isolated from surgical resected tumor specimens obtained from the patients suffered from stage II or stage III colon adenocarcinoma, respectively. Both patients were diagnosed with moderately differentiated tumors. NF1/NF2 were derived from para-cancer normal mucosa of another two patients with colon cancer. None of them received radiotherapy or chemotherapy prior to surgical resection. In brief, fresh tumor specimens and adjacent normal mucosal tissues were minced into small pieces and washed with PBS (supplement with 10% penicillin/streptomycin) 3 times. Samples were then transferred into a gentleman’s C Tube and digested with the following solution (5 mL DMEM + 100 μl Enzyme H + 50 μl Enzyme R + 12.5 μl Enzyme A) using gentleMACS™ Octo Dissociator for 1 h. After incubation, cells were collected by centrifuging at 2000 rpm for 7 min and passed through a 70 μm cell strainer. Erythrocytes were depleted by 1 × RBC Lysis Buffer. Collected cells were cultured in the DMEM medium (supplement with 10%FBS, 1%NEAA, 2.5 μg/ml amphotericin B (1:5000), 200 μg/ml gentamycin (1:500), 1%PS, 0.1%CIP, 4 μg/ml insulin) [22, 51]. After 48 h, unattached cells and debris were removed. Cells left were washed and plated at high density in DMEM supplemented with 10%FBS. Cell morphology was observed under phase-contrast microscopy. Total RNA was extracted and qRT-PCR analysis of typical CAFs-associated markers including Fibronectin, α-SMA, and P4HA1 was performed to verify the establishment of fibroblasts.

### RNA sequencing and data analysis

To search for essential genes involved in regulating metastasis of CRC which are associated with cancer-associated fibroblasts, RNA sequencing was performed using 4 primary tumors (PT) and 3 liver metastases (LM) from CRC patients as well as cancer cells which were separately co-cultured with CAFs or not. In brief, total RNA was extracted using a mini RNA isolation kit (cat. no.74106; Qiagen, Germany) and transcribed to cDNA with random primers. The cDNA was ligated to Illumina sequencing adaptors and sequenced on the Illumina NovaSeq6000 by Gene Denovo Biotechnology Co. (Guangzhou, China). Gene expression was defined by FPKM value (fragments per kilobase of exon per million fragments mapped) using RSEM software. Bioinformatics analysis was performed using Omicsmart, a dynamic and interactive online platform for data analysis (https://www.omicsmart.com). A log_2_ (fold change) >2 and a false discovery rate (FDR) < 0.01 were used as cut-off thresholds.

### Clinical specimens and immunohistochemistry (IHC)

All tumor tissue specimens were obtained from the Tissue Bank with approval from the Human Medical Ethics Committee of Sun Yat-Sen University and with written informed consent from all patients for the use of their data. Pathological diagnoses were performed by experienced pathologists. Clinicopathologic parameters and 5 years of follow-up information were obtained from the Follow-up Database.

Paraffin-embedded specimens of 205 colorectal primary tumors and 15 liver metastatic tumors were selected for IHC. Briefly, slides were deparaffinized, rehydrated and antigens were retrieved by EDTA antigen retrieval solution (PH = 9.0) (Service bio, China) using microwave. Samples were then incubated with primary antibody against LRG1(1:100, Abcam, USA) or α-SMA (1:7500, CST, USA) overnight at 4 °C, followed by incubation with anti-rabbit/mouse Labeled Polymer (Dako, CA). DAB reagent kit (G1212-200T, Service bio China) was used as the chromogen and hematoxylin was used as counterstain. LRG1 and α-SMA expression were quantified based on the intensity of staining and the percentage of positive tumor cells. In brief, the proportion of positive cells was estimated and given a score ranging from 1 to 4 (1, less than 5%; 2, 5–25%; 3, 26–50%; 4, > 51%). The average intensity of the positively stained cells was also given a score on a scale from 1 to 4 (1, no staining; 2, weak staining; 3, moderate staining; 4, strong staining). A final IHC score of each TMA spot was then calculated via multiplying the positive percentage score by the intensity score [52]. The high or low expression of target proteins was defined by X-tile software (X-tile 3.6.1).

### Reagents

Recombinant IL-6 and IL-8 were purchased from Peprotech (Rocky Hill, NJ), recombinant TGF-β2 was purchased from Invitrogen (Waltham, MA), and recombinant IL-1β was purchased from NovoProtein (catalog number: CC93-10μg). The HO-3867 (catalog number: HY-100453), Pacritinib (catalog number: HY-16379), Tocilizumab (catalog number: HY-9917), SB431542(catalog number: HY-10431/CS-0135) were purchased from MCE. IL-6 neutralizing antibody (catalog number: MAB206) was purchased from R&D Systems (Minneapolis, MN). Cells were treated with Pacritinib (1 μmol/ml), HO-3867 (3 μmol/ml), Tocilizumab (2.5 ng/ml) or SB-431542 (0.25μmol/ml) 4 h before conditioned medium treatment. IL-6 antibody (2.5 ng/ml) was used to neutralize IL-6 in the CM.

### Cell culture

All cell lines were purchased from ATCC and mycoplasma testing was performed at the start of the project. DLD-1, HCT-116, Plat-A, and HEK 293 T were cultured in DMEM medium. CT-26 was cultured in RPMI 1640 medium. All media were supplemented with 10% fetal bovine serum (FBS) and 1% Penicillin/Streptomycin (PS). All cell lines were maintained at 37 °C in a humidified atmosphere with 5% CO2.

### RNA extraction and qRT-PCR

Total RNA was extracted using TRIzol reagent (Invitrogen, Thermo Fisher Scientific, and Waltham, MA, USA) or Direct-zol RNA Miniprep (Zymo Research, Irvine, CA; catalog number: R2050) according to manufacturer’s instruction. cDNA was reversely transcribed using qPCR RT Master Mix (catalog number: 00992040; Thermo Fisher Scientific, USA) and subjected to PCR amplification using SYBR Green (catalog number: 1725125; Biorad, USA) on a Light Cycler 480 instrument (Roche, Basel, Switzerland). Relative expression of each gene was normalized to GAPDH. Primers used for qRT–PCR were summarized in supplementary Table 1.

### Western blot

Cell pellets were lysed with RIPA buffer containing protease/phosphatase inhibitor cocktail (catalog number: 4693116001; Roche, Germany) and protein concentration was measured by BCA kit. Equal amounts of total protein were subjected to SDS-PAGE, followed by blocking with 5 % fat-free milk. Primary antibodies were incubated at 4 °C overnight, followed by secondary antibodies for 40 min at room temperature. All blots were developed using a chemiluminescence kit (SuperSignal ECL Kit, Thermo Fisher, USA). The following antibodies were used: anti-LRG1 (catalog number: #178698, 1:1000 dilution) was purchased from Abcam (Cambridge, MA). Anti-Tubulin (catalog number: #T4026S, 1:1000 dilution) was purchased from Sigma. Anti-phospho-STAT3(y705) (catalog number: #9541 S, 1:1000 dilution), anti-STAT3 (catalog number: #9139 S, 1:1000 dilution), anti-N-cadherin (catalog number: #13116t, 1:1000 dilution), anti-E-cadherin (catalog number: #3195 T, 1:1000 dilution), and anti-Slug (catalog number: #C19G7, 1:1000 dilution), Anti-phospho-Smad1/5(Ser463/465) (catalog number: #9516, 1:1000 dilution) were purchased from Cell Signaling Technology (Danvers, MA). Anti-Twist1 (catalog number: #abs131127-50μg, 1:1000 dilution) was purchased from Absin (China).

### Small interfering RNA silencing

Two independent siRNA targeting STAT3 or negative control siRNA (GenePharma, ShangHai, China) was transfected into DLD-1 using RNA IMAX transfection reagent (catalog number: 13778030; Thermo Fisher Scientific, USA) according to the manufacturer’s instructions. After incubation for 6 h, cells were replaced with fresh culture medium. 24 h later, cells were treated with conditioned medium or not and subjected to further experiments. The siRNA sequences targeting STAT3 are as follows: #1 5ʹ-AACUUCAGACCCGUCAACAAA-3ʹ; #2 5ʹ-AACAUCUGCCUAGAUCGGCUA-3ʹ.

### Generation of stable cell lines

To establish stable cell lines with silencing or ectopic expression of LRG1, plasmids containing two different shLRG1 sequences or full-length LRG1 cDNA were constructed separately using pLKO.1-puro or PMN-puro empty vector. Virus particles were produced in 293 T with help of packaging plasmids or Plat-A as described previously [53]. The sequences of shRNA targeting LRG1 are listed below: #1: sense,5ʹ-GATGTTTTCCCAGAATGAC-3ʹ, antisense,5ʹ-GTCATTCTGGGAAAACATC-3ʹ; #2: sense,5ʹ-GCAATTAGAACGGCTACAT-3ʹ, antisense,5ʹ-ATGTAGCCGTTCTAATTGC-3ʹ.

Primers used for amplifying full-length cDNA of human LRG1 are as follows:

Forward primer 5ʹ-CGGAATTCATGTCCTCTTGGAGCAGAC-3ʹ, reverse primer 5ʹ-CGCAATTGATGTCCTCTTGGAGCAGAC-3ʹ.

### Co-culture system and conditioned medium

For co-cultured system, the CAFs (2×10^4^ cells) were seeded in the collagen-coated 24-well chamber insert (8 µm, Corning Falcon, catalog number: 353097), while DLD-1or HCT-116 (5 × 10^4^ cells) were seeded in the flat bottom 24-well plate (Corning Falcon, catalog number: 353504). After separate culture for one day, the insert with CAFs was put into 24-well plate containing cancer cell lines for indicated period to establish CAFs-cancer cells separate co-culture model. The RNA from cancer cells was extracted for RNA-seq or quantitative RT-PCR assay.

To obtain conditioned medium (CM), CAFs (1 × 10^5^ cells) or NF (1 × 10^5^ cells) were seeded in the collagen-coated flat bottom 6-well plate and maintained in DMEM medium (Gibco) containing 10% FBS (Gibco) for 48 h. The medium was harvested and centrifuged at 2,000 rpm for 5 min. The clear supernatant was then collected as CM. DLD-1 or HCT-116 was seeded in the flat bottom 12-well plate or 6-well plate and treated with CAF-CM or NF-CM for 24-48 h, followed by quantitative RT-PCR assay or western blot.

### Cell proliferation, migration, and invasion assay

Exponentially growing cells were seeded into 96-well plates at a density of 5000 cells per well. Cell viability was assessed using cell Counting Kit-8 (CCK-8) (Beyotime, Jiangsu, China) every day and plotted as a growth curve.

A wound-healing assay was used to examine the migration capacity of tumor cells in vitro. DLD-1 (1 × 10^6^) and HCT-116 (1 × 10^6^) cells stably expressing shCtrl or sh*LRG1* with control medium or conditioned medium pre-treatment or not were seeded in a 12-well plate. Control or conditioned medium with low serum (2%) was used. The next day, the monolayer cells were scratched with a 200-ul pipette tip evenly to generate a wound. To track wound closure rate, images from five random fields were taken under phase-contrast microscopy every 12 h. The Images taken at 24 h were used for wound healing rate calculation by Image J software.

Trans-well assay was performed to analyze the invasive capacity of tumor cells (Falcon, BD Bioscience). DLD-1 (4 × 10^4^) and HCT-116 (8 × 10^4^) cells stably expressing shCtrl or shLRG1 were seeded onto upper surface of inserts pre-coated with Matrigel (1:20, cat. no. 356234; Corning, NY, USA). The top chamber was then filled with 100 μl serum-free medium and complete medium with 10% FBS was added into the bottom as chemotaxis. To assess the effect of CAFs on invasion, CM was added into the bottom chamber. After incubation for 24 h, cells on the lower surface of the insert were fixed with 4% paraformaldehyde and stained with 0.1% crystal violet (cat. no. 0424A17; League, Beijing, China). Images of invaded cells at 24 h were captured from five random fields per condition with a microscopy. The invasion rate was calculated with Image J software.

### Tumorsphere formation assay

Active growing cells were treated with 0.05% trypsin for 5 min then passed through 0.4 mm cell strainer, to achieve single cell suspension. Cells were plated (DLD-1/HCT-116 5 × 10^3^) in six-well ultra-low attachment plates (Corning, Corning, NY, cat: CLS3471) in Mammocult medium (Stem Cell Technologies, Vancouver, BC, Canada) supplemented with fresh hydrocortisone (0.5 mg/ml) and heparin (1:500) which was subsequently mixed with control medium or conditioned medium in a 1:1 ratio. Tumorspheres were cultured for 7 days before being counted and photographed. Analysis of the number of tumorspheres with diameter>0.1 cm was considered.

### Animal model of splenic liver metastasis

The animal experiment was approved by the Animal Research Committee of the Sixth Affiliated Hospital of Sun Yat-sen University and carried out in accordance with its guidelines. Six to eight-week-old nude mice (male) were purchased from Gempharmatech Co., Ltd. (China). Three groups of cells: DLD-1(shCtrl), DLD-1(shCtrl)+CM and DLD-1(sh*LRG1*) +CM were used for intra-splenic injection. ShCtrl and sh*LRG1* represent the cells expressing control or *LRG1* shRNA, and CM means the cells were pre-treated with conditioned medium (CM) from CAF1 for 10 days before injection. Nude mice were anesthetized with isoflurane by using mouse anesthesia apparatus. A small left abdominal flank incision was made and the spleen was exteriorized for the intra-splenic injection. Tumor cells (2 × 10^6^) in 50 μl phosphate-buffered saline solution were injected into the spleen using a 30-gauge needle. To prevent tumor cell leakage and bleeding, a cotton swab containing 75% ethanol were firmly placed on the spleen injection site for 1 min. The injected spleen was returned to the abdomen and the wound was sutured with 6-0 black silk. Four weeks later, the mice were sacrificed and tumor formation in spleen and liver metastasis were examined. Hematoxylin and eosin (H&E) staining was performed in livers taken from all three groups.

### Dual-luciferase report assay

To investigate the effect of STAT3 on activation of *LRG1* promoter, Dual-luciferase reporter assay was performed using DLD-1 cells treated with CM from CAFs or not. The DLD-1 cells (1 × 10^5^ cells) were seeded in a 24-well plate and transfected with plasmids as follows: pGL3 (control vector); full-length LRG1 promoter containing two potential STAT3 binding site-BS1 and BS2 (pGL3-LRG1-FL); promoter region of LRG1 with BS1 alone (pGL3-LRG1-BS1), promoter region of LRG1 with BS2 alone (pGL3-LRG1-BS2) and promoter region of LRG1-BS1 with BS1 deletion (pGL3-LRG1-BS1 mutation). pRL null was used as an internal control and co-transfected with indicated plasmids at a ratio of 1:100. Forty-eight hours after transfection, the luciferase activity was measured using Dual-Luciferase Reporter Assay System Kit (E1910, Promega) according to the manufacturer’s instructions. Primers for amplifying promoter regions of LRG1 were shown in Supplementary Table 2.

### ChIP-PCR

DLD-1 (2 × 10^6^ cells) were cross-linked in 3.7% formaldehyde for 10 min and quenched with 2 M glycine solution. Then the cells were lysed with SDS lysis buffer (50 mM Tris-HCl pH8.0, 10 mM EDTA, 1% SDS) and chromatin was sheared to fragments around 300 ~ 500 bps by sonication [54]. Lysate was pre-cleared with protein A/G agarose beads (Santa Cruz Biotech, Santa Cruz, CA, USA) for 4 h and subsequently incubated with IgG (Santa Cruz, catalog number: #sc-2027, 1:100) or antibody against stat3 (Cell Signaling, catalog number: #9139, 1:100) overnight. After washing for three times, the immunoprecipitated DNA was eluted using SDS-Elusion Buffer (50 mM Tris-HCl pH7.5, 10 mM EDTA, 1% SDS) and cross-link was reversed by TE Buffer (10 mM Tris-HCl pH7.5, 1 mM EDTA). DNA was purified by phenol-chloroform extraction and ethanol precipitation. The primer sequences are provided in supplementary Table 2.

### Statistical analysis

All in vitro experiments were repeated at least three times unless stated otherwise, and the data are shown as mean ± SD. For all in vitro experiments, P-value was calculated by either a two-tailed Student’s *t* test or one-way ANOVA. The survival curve of clinical patients was plotted using Kaplan–Meier analysis. The association between LRG1 staining and the clinicopathologic features of CRC patients was examined by Chi-square tests. All tests were performed using the software GraphPad Prism. Significance was denoted in the figures as *p < 0.05, **p < 0.01, ***p < 0.001, and ****p < 0.0001, ns for no significance.

## Supplementary information

Supplemenatal materials

Supplemental table 1

Supplemental table 2

Reproducibility checklist

## Data Availability

The microarray data generated in this study has been deposited in the Gene Expression Omnibus (GEO) under accession numbers GSE179979. The online dataset GSE14297, GSE50760, and GSE2082 used in this study are available in GEO dataset. The CPTAC protein data used in this study are available in website of CPTAC (Clinical Proteomic Tumor Analysis Consortium) data portal (https://proteomics.cancer.gov/data-portal).
