# Supplementary material for: Colorectal cancer-associated fibroblasts promote metastasis by up-regulating LRG1 through stromal IL-6/STAT3 signaling
Source: Cell Death Dis. 2021 Dec 20;13(1):16. doi: 10.1038/s41419-021-04461-6 (PMC8688517; doi:10.1038/s41419-021-04461-6)
Supplement: Supplementary file 1 — Supplemenatal materials [file 41419_2021_4461_MOESM1_ESM.pdf]

Supplementary Fig1

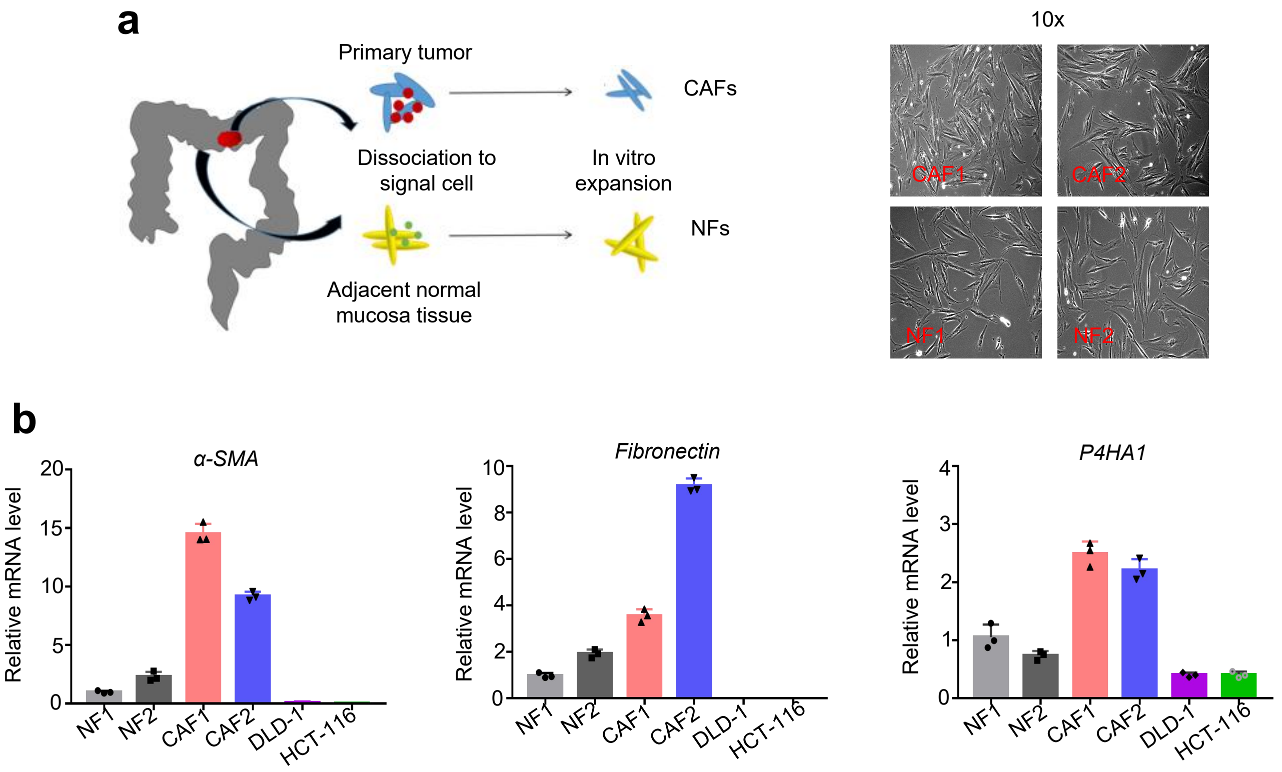

Supplementary Figure 1. Establishment of patient-derived fibroblasts

a. Scheme depicting the process of fibroblasts extraction (Left). Representative images showing morphology of extracted fibroblasts observed under phase-contrast microscopy (right).

b. QRT-PCR analysis of CAF markers including α-SMA, Fibronectin, and P4HA1 in NFs, CAFs and CRC cell lines.

Supplementary Fig2

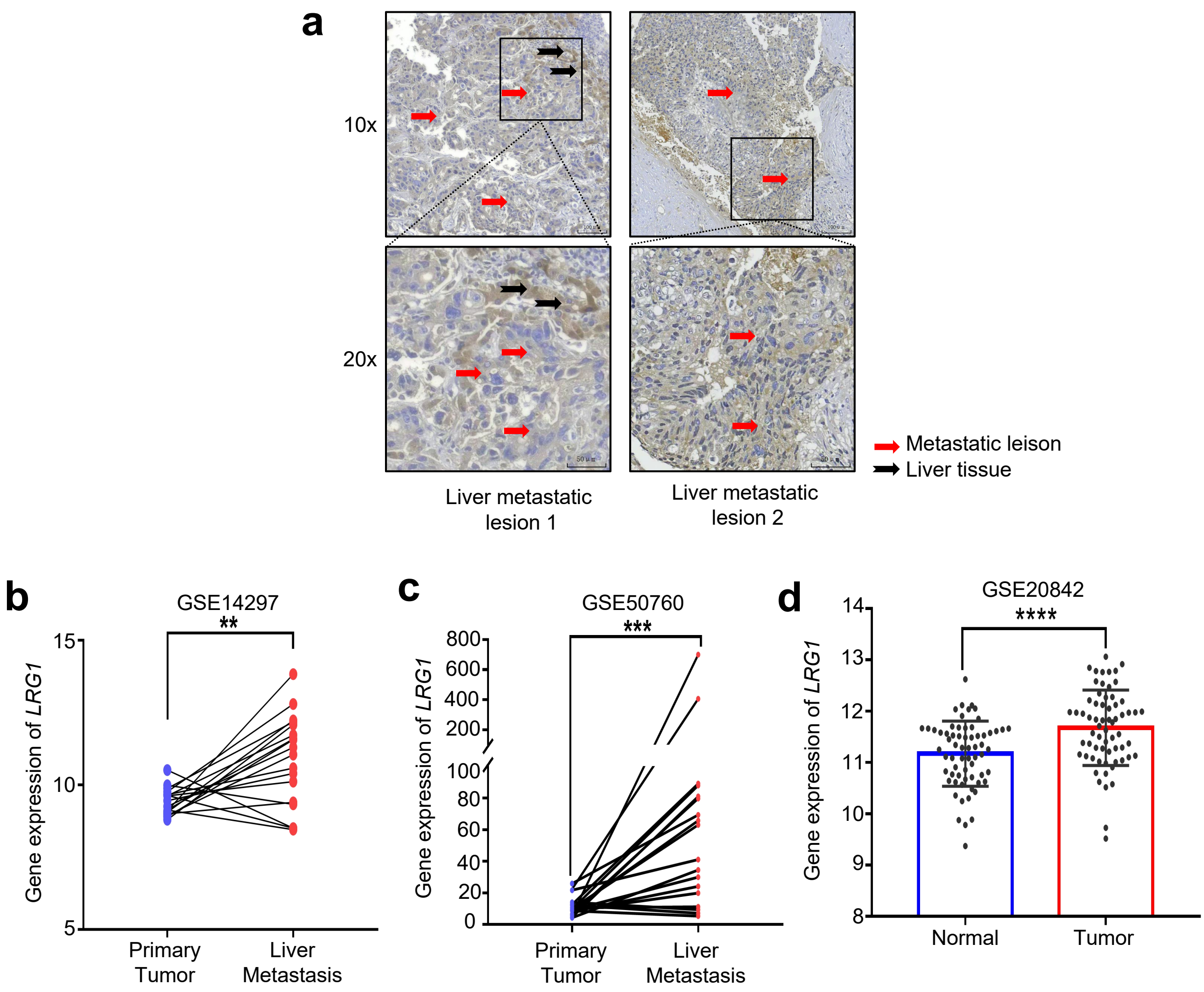

Supplementary Figure 2. LRG1 shows higher expression in metastatic CRC lesions

a. Representative IHC staining of LRG1 in tumor sections and liver tissue from liver metastatic CRC patients (Scale bar, 50μm). The red arrows represent liver metastatic lesion, and the black arrows represent liver tissue.

b&c. Analysis of LRG1 expression comparing CRC liver metastatic tissues to primary tumor tissues based on microarray data from GSE14297 and GSE50760 (both are paired samples)

d. Analysis of LRG1 expression in CRC primary tumor tissues versus normal tissues based on microarray data from GSE20842. (N stands for normal intestinal mucosal tissue; T stands for CRC primary tumor).

Error bars represent SD; n= 3. ***$P < 0.01$, ****$P < 0.001$, *****$P < 0.0001$.

Supplementary Fig3

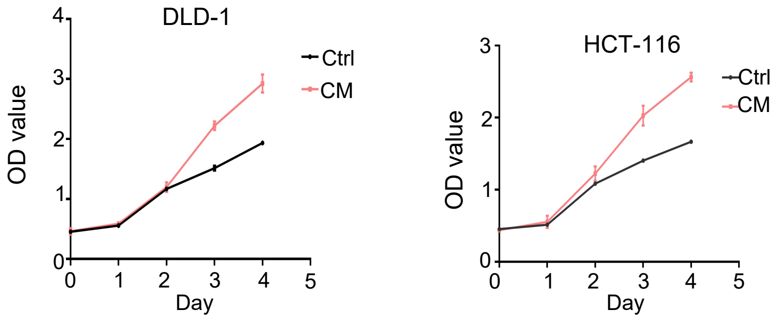

Supplementary Figure 3. CAF conditioned medium promotes cell proliferation
Growth curves of DLD-1 and HCT-116 treated with control medium or conditioned medium from CAF1 were measured with CCK8 kit.

Supplementary Fig4

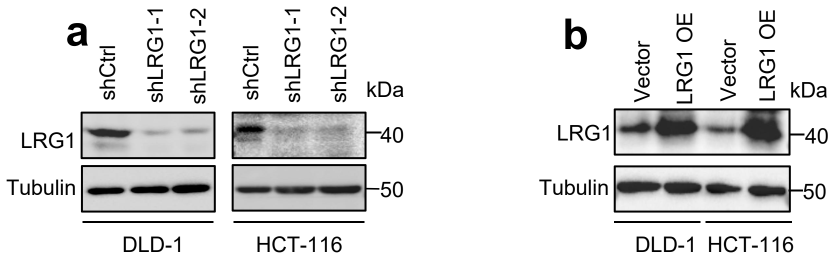

Supplementary Figure 4. LRG1 expression in *LRG1*-knockdown or -overexpression cells

a. Silencing of LRG1 in DLD-1 and HCT-116 was verified by western blot.

b. Ectopic expression of LRG1 in DLD-1 and HCT-116 was verified by western blot.

Supplementary Fig5

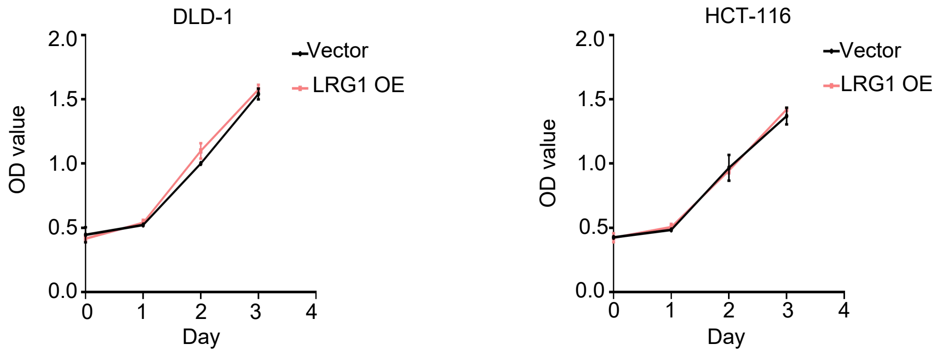

Supplementary Figure 5. Cell proliferation of cells with empty vector or LRG1 overexpression

Growth curves of DLD-1 and HCT-116 with empty vector or LRG1 overexpression were measured by CCK8 kit.

Supplementary Fig6

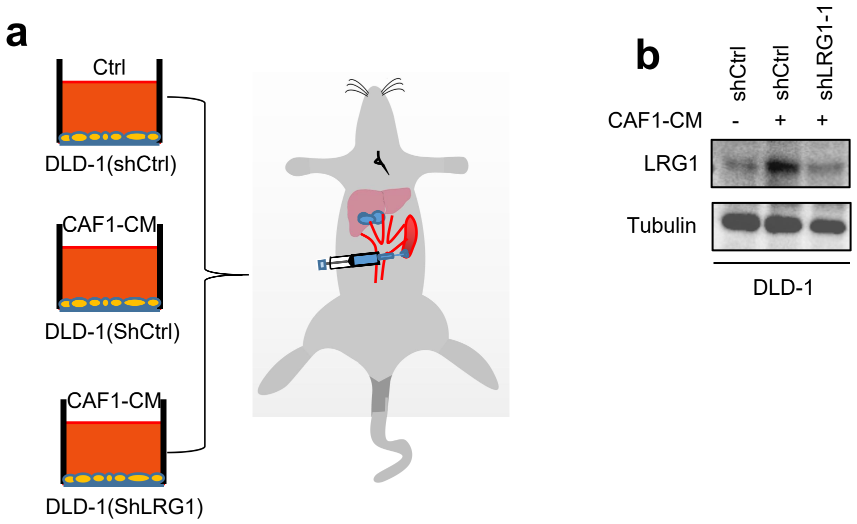

Supplementary Figure 6. Animal model of transsplenic liver metastasis

a. Scheme diagram depicting the animal model of transsplenic liver metastasis.

b. Expression of LRG1 in DLD1 expressing shctrl or sh*LRG-1,* which was cultured with control medium or CM from CAFs as indicated, was measured by western blot.

Supplementary Fig7

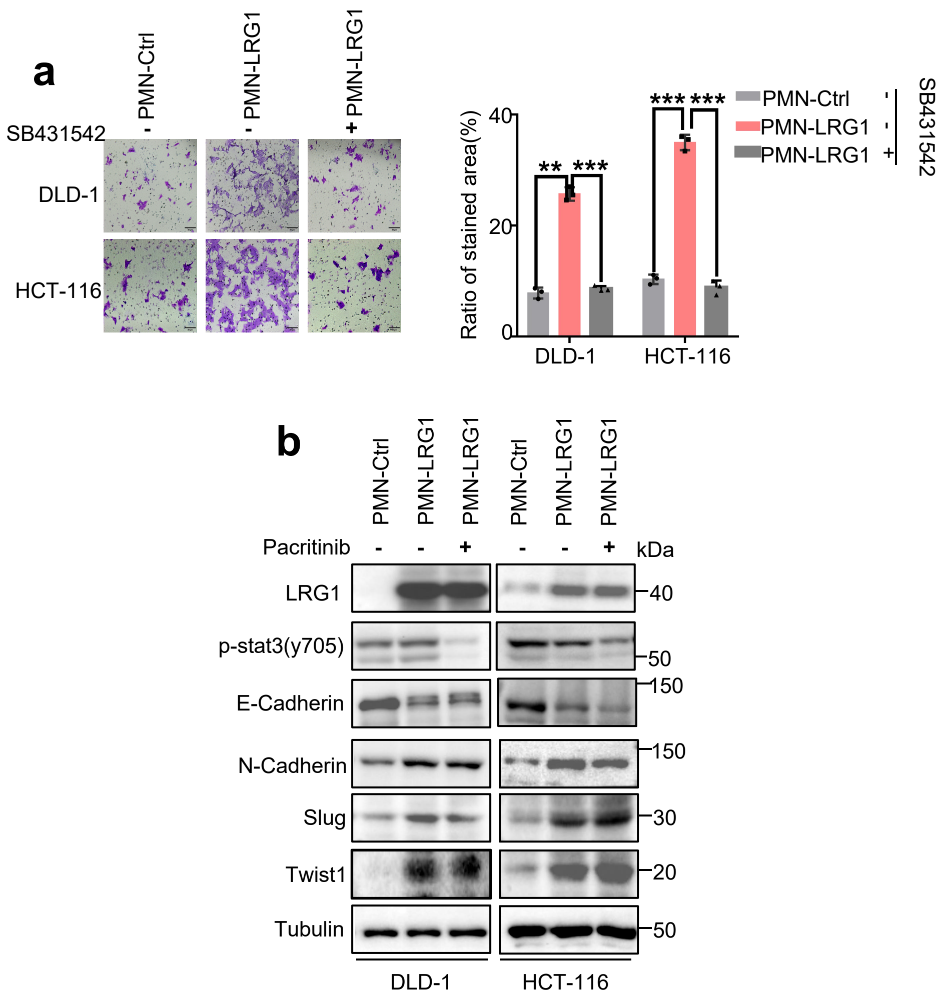

Supplementary Figure 7. LRG1-induced EMT dependents on Smad1/5 but not STAT3 activation

a. DLD-1 and HCT-116 with empty vector or LRG1 overexpression were treated with SB431542, then their invasive capability was assessed by Boyden chamber assay. Left: Representative images showing invaded DLD-1 and HCT-116 cells. Right: Graphs showing statistical analysis of the percentage of invasive cells area at 24h.

b. DLD1 and HCT-116 with empty vector or LRG1 overexpression were treated with Pacritinib, a JAK2 inhibitor. Expression of LRG1, phosphorylated stat3(y705) and EMT-associated markers were measured by western blot.

Error bars represent SD; n= 3. $^{**}P < 0.01$, $^{***}P < 0.001$.
